# Limit properties for ratios of order statistics from exponentials

**DOI:** 10.1186/s13660-016-1287-6

**Published:** 2017-01-06

**Authors:** Yong Zhang, Xue Ding

**Affiliations:** College of Mathematics, Jilin University, Changchun, 130012 P.R. China

**Keywords:** exponential distribution, order statistics, strong law of large numbers, central limit theorem, law of iterated logarithm

## Abstract

In this paper, we study the limit properties of the ratio for order statistics based on samples from an exponential distribution and obtain the expression of the density functions, the existence of the moments, the strong law of large numbers for $R_{nij}$ with $1\leq i< j< m_{n}=m$. We also discuss other limit theorems such as the central limit theorem, the law of iterated logarithm, the moderate deviation principle, the almost sure central limit theorem for self-normalized sums of $R_{nij}$ with $2\leq i< j< m_{n}=m$.

## Introduction and main results

Throughout this note, let $\{X_{ni}, 1\leq i\leq m_{n}\}$ be a sequence of independent exponential random variable with mean $\lambda_{n}$, let $\{X_{n},n\geq1\}=:\{(X_{ni}, 1\leq i\leq m_{n}),n\geq1\}$ be an independent random sequence, where $\{m_{n}\geq2\} $ denotes the sample size. Denote the order statistics be $X_{n(1)}\leq X_{n(2)} \leq\cdots\leq X_{n(m_{n})}$, and the ratios of those order statistics

$$R_{nij}=\frac{X_{n(j)}}{X_{n(i)}}, \quad 1\leq i< j\leq m_{n}. $$

As we know, the exponential distribution can describe the lifetimes of the equipment, and the ratios $R_{nij}$ can measure the stability of equipment, it shows whether or not our system is stable. Adler [1] established the strong law of the ratio $R_{n1j}$ for $j\geq2$ with fixed sample size $m_{n}=m$, and the strong law of $R_{n12}$ for $m_{n}\to\infty$ as follows.

### Theorem A

*For fixed sample size* $m_{n}=m$ *and all* $\alpha>-2$, $2\leq j\leq m$, *we know*

$$ \lim_{N\to\infty}\frac{1}{(\log N)^{\alpha+2}}\sum_{n=1}^{N} \frac{(\log n)^{\alpha}}{n}R_{n1j}=\frac{ m!}{(j-2)!(m-j)!(\alpha+2)}\sum _{l=0}^{j-2}C_{j-2}^{l} \frac{(-1)^{j-l-2}}{(m-l-1)^{2}} \quad \textit{a.s.} $$

*For* $m_{n}\to\infty$ *and all* $\alpha>-2$,

$$ \lim_{N\to\infty}\frac{1}{(\log N)^{\alpha+2}}\sum_{n=1}^{N} \frac{(\log n)^{\alpha}}{n}R_{n12}=\frac{1}{\alpha+2}\quad \textit{a.s.} $$

Later on, Miao *et al.* [2] proved the central limit theorem and the almost sure central limit for $R_{n23}$ with fixed sample size, we state their results as the following theorem.

### Theorem B

*For fixed sample size* $m_{n}=m$,

$$\begin{aligned}& \frac{1}{\eta_{N}}\sum_{n=1}^{N}(R_{n23}-ER_{n23}) \stackrel{\mathrm {{D}}}{\rightarrow}N(0,1) \quad \textit{as }N\to\infty, \\& \lim_{N\to\infty}\frac{1}{\log N}\sum_{n=1}^{N} \frac{1}{n}I\Biggl\{ \frac{1}{\eta _{N}}\sum_{n=1}^{N}(R_{n23}-ER_{n23}) \leq x\Biggr\} =\Phi(x)\quad \textit{a.s.} \end{aligned}$$

*for all* $X\in R$, *where* $\Phi(\cdot)$ *denotes the distribution function of* $N(0,1)$, $\eta_{n}=1\vee\sup\{r>0; nL(r)\geq r^{2}\}$, $L(r)=ER_{n23}^{2}I\{|R_{n23}|\leq r\}$.

In this paper, we will make a further study on the limit properties of $R_{nij}$. In the next section, firstly, we give the expression of the density functions of $R_{nij}$ for all $1\leq i< j< m_{n}$, it is more interesting that the density function is free of the sample mean $\lambda_{n}$, this allows us to change the equipment from sample to sample as long as the underlying distribution remains an exponential. Also we discuss the existence of the moments for fixed sample size $m_{n}=m$. Secondly, we establish the strong law of large number for $R_{nij}$ with $1=i< j< m$ and $2\leq i< j< m$, respectively. At last we give some limit theorems such as the central limit theorem, the law of iterated logarithm, the moderate deviation principle, the almost sure central limit theorem for self-normalized sums of $R_{nij}$ with $2\leq i< j< m$.

In the following, C denotes a positive constant, which may take different values whenever it appears in different expressions. $a_{n}\sim b_{n}$ means that $a_{n}/b_{n}\rightarrow1$ as $n\rightarrow \infty$.

## Main results and proofs

### Density functions and moments of $R_{nij}$

The first theorem gives the expression of the density functions.

#### Theorem 2.1

*For* $1\leq i< j\leq m_{n}$, *the density function of the ratios* $R_{nij}$ *is*

2.1 $$ f_{nij}(r)=\frac{m_{n}!}{(i-1)!(j-i-1)!(m_{n}-j)!}\sum _{k=0}^{i-1}\sum_{l=0}^{j-i-1} \frac {(-1)^{j-k-l-2}C_{i-1}^{k}C_{j-i-1}^{l}}{[i-k+l+r(m_{n}-i-l)]^{2}}I\{r>1\}. $$

#### Proof

It is easy to check that the joint density function of $X_{n(i)}$ and $X_{n(j)}$ is

$$\begin{aligned} f(x_{i},x_{j}) =&\frac{m_{n}!}{(i-1)!(j-i-1)!(m_{n}-j)!}\frac{1}{\lambda _{n}^{2}} \bigl[1-e^{-x_{i}/{\lambda_{n}}}\bigr]^{i-1}\bigl[e^{-x_{i}/{\lambda _{n}}}-e^{-x_{j}/{\lambda_{n}}} \bigr]^{j-i-1} \\ &{}\cdot e^{-x_{i}/{\lambda_{n}}}e^{-(m_{n}-j+1)x_{j}/{\lambda_{n}}}I\{0< x_{i}< x_{j} \}. \end{aligned}$$

Let $w=x_{i}$, $r=x_{j}/{x_{i}}$, then the Jacobian is *w*, so the joint density function of *w* and *r* is

$$\begin{aligned} f(w,r) =&\frac{m_{n}!}{(i-1)!(j-i-1)!(m_{n}-j)!}\frac{w}{\lambda _{n}^{2}}\bigl[1-e^{-w/{\lambda_{n}}} \bigr]^{i-1}\bigl[e^{-w/{\lambda_{n}}}-e^{-rw/{\lambda _{n}}}\bigr]^{j-i-1} \\ &{}\cdot e^{-w/{\lambda_{n}}}e^{-(m_{n}-j+1)rw/{\lambda_{n}}}I\{w>0, r>1\}. \end{aligned}$$

Therefore the density function of $R_{nij}$ is

$$\begin{aligned}& f_{nij}(r)= \int_{0}^{\infty}f(w,r)\, dw \\& \quad = \frac{m_{n}!}{(i-1)!(j-i-1)!(m_{n}-j)!}\frac{1}{\lambda_{n}^{2}} \sum_{k=0}^{i-1} \sum_{l=0}^{j-i-1}(-1)^{j-k-l-2}C_{i-1}^{k}C_{j-i-1}^{l} \\& \qquad {} \cdot \int_{0}^{\infty}we^{-(i-k+l)w/{\lambda _{n}}}e^{-(m_{n}-i-l)rw/{\lambda_{n}}}\,dw \\& \quad = \frac{m_{n}!}{(i-1)!(j-i-1)!(m_{n}-j)!} \sum_{k=0}^{i-1} \sum_{l=0}^{j-i-1}(-1)^{j-k-l-2}C_{i-1}^{k}C_{j-i-1}^{l} \\& \qquad {} \cdot \int_{0}^{\infty}te^{-[(i-k+l)+r(m_{n}-i-l)]t}dt \\& \quad = \frac{m_{n}!}{(i-1)!(j-i-1)!(m_{n}-j)!}\sum_{k=0}^{i-1} \sum_{l=0}^{j-i-1}(-1)^{j-k-l-2} \frac{C_{i-1}^{k}C_{j-i-1}^{l}}{[i-k+l+r(m_{n}-i-l)]^{2}} . \end{aligned}$$

 □

The next theorem treats the moments of $R_{nij}$ with fixed sample size $m_{n}=m$.

#### Theorem 2.2

*For fixed sample size* $m_{n}=m$ *and* $1=i< j\leq m$, *we know*

$$ ER_{n1j}^{\gamma}=\left \{ \textstyle\begin{array}{l@{\quad}l} {< }\infty, & 0< \gamma< 1, \\ {=}\infty, & \gamma\geq1, \end{array}\displaystyle \right . $$

*and with* $2\leq i< j\leq m$,

$$ ER_{nij}^{\gamma}=\left \{ \textstyle\begin{array}{l@{\quad}l} {< }\infty, & 0< \gamma< 2, \\ {=}\infty, & \gamma\geq2. \end{array}\displaystyle \right . $$

*Let* $L(r)=E(R_{nij}-ER_{nij})^{2}I\{|R_{nij}-ER_{nij}|\leq r\}$, $2\leq i< j\leq m$, *then* $L(r)$ *is a slowly varying function at* ∞.

#### Proof

For $1=i< j\leq m$, by (2.1), it is easy to check that

$$\begin{aligned} f_{n1j}(r) =&\frac{m!}{(j-2)!(m-j)!}\sum_{l=0}^{j-2}(-1)^{j-l-2}C_{j-2}^{l} \frac{1}{[1+l+r(m-l-1)]^{2}} \\ \sim&\frac{c_{m,j}}{r^{2}}\quad \mbox{as }r \to\infty, \end{aligned}$$

where $c_{m,j}$ is a constant depend only on *m* and *j*. Obviously the *γ*-order moment is finite for $0<\gamma<1$ and is infinite for $\gamma\geq1$.

For $2\leq i< j\leq m$, similarly we can obtain $f_{nij}(r)\sim\frac{d_{m,i,j}}{r^{3}}$, where $d_{m,i,j}$ is a constant depend only on *m*, *i* and *j*, so the *γ*-order moment is finite for $0<\gamma<2$ and is infinite for $\gamma\geq2$. Furthermore it is not difficult to verify that $L_{1}(r)=E R_{nij}^{2}I\{|R_{nij}|\leq r\}$ varies slowly at ∞, then by the fact that if $L(x)=E|X|^{2}I\{|X|\leq x\}$ is a slowly varying function at ∞, then $L_{a}(x)=E|X-a|^{2}I\{|X-a|\leq x\}$ also varies slowly at ∞ for any $a\in R$, the proof is completed. □

#### Remark 2.3

Miao *et al.* [2] obtained the density function for $R_{n2j}$ for fixed sample size $m_{n}=m$, they also proved that the expectation of $R_{n2j}$ is finite and the truncated second moment is slowly varying at ∞. Adler [1] also claimed that all the $R_{n1j}$ have infinite expectations for fixed sample size, so our theorems extended their results.

### Strong law of large numbers of $R_{nij}$

From our assumptions, we know that $\{R_{nij},n\geq1\}$ is an independent sequence with the same distribution for fixed sample size $m_{n}=m$. As Theorem 2.2 states that the $R_{n1j}$ do not have the expectation, so the strong law of large numbers with them is not typical. Here we give the weighted strong law of large number as follows. At first, we list the following lemma, that is, Theorem 2.6 from De la Peña *et al.* [3], which will be used in the proof.

#### Lemma 2.4

*Let* $\{X_{n},n\geq1\}$ *be a sequence of independent random variables*, *denote* $S_{n}=\sum_{i=1}^{n}X_{i}$, *if* $b_{n}\nearrow\infty$, *and* $\sum_{i=1}^{\infty}\operatorname{Var}(X_{i})/{b_{i}^{2}}<\infty$, *then* $(S_{n}-ES_{n})/{b_{n}}\to 0$ *a*.*s*.

#### Theorem 2.5

*Let* $\{a_{n},n\geq1\}$ *be a sequence of positive real numbers and* $\{ b_{n},n\geq1\}$ *be a sequence of nondecreasing positive real numbers with* $\lim_{n\to\infty}b_{n}=\infty$ *and*

2.2 $$\begin{aligned}& \sum_{n=1}^{\infty} \frac{a_{n}}{b_{n}}< \infty, \end{aligned}$$

2.3 $$\begin{aligned}& \lim_{N\to\infty}\frac{1}{b_{N}}\sum _{n=1}^{N}a_{n}\log\biggl(\frac {b_{n}}{a_{n}} \biggr)=\lambda\in[0,\infty). \end{aligned}$$

*Then*, *for the fixed sample size* $m_{n}=m$ *and* $2\leq j\leq m$, *we have*

2.4 $$ \lim_{N\to\infty} \frac{1}{b_{N}}\sum _{n=1}^{N}a_{n}R_{n1j}= \frac{\lambda m!}{(j-2)!(m-j)!}\sum_{l=0}^{j-2}C_{j-2}^{l} \frac{(-1)^{j-l-2}}{(m-l-1)^{2}}\quad \textit{a.s.} $$

*For* $m_{n}\to\infty$,

2.5 $$ \lim_{N\to\infty} \frac{1}{b_{N}}\sum _{n=1}^{N}a_{n}R_{n12} =\lambda \quad \textit{a.s.} $$

#### Proof

By (2.2) we get $c_{n}=b_{n}/{a_{n}}\to\infty$, so without loss of generality we assume that $c_{n}\geq1$ for any $n\geq1$. Notice that

2.6 $$\begin{aligned} \frac{1}{b_{N}}\sum_{n=1}^{N}a_{n}R_{n1j} =& \frac{1}{b_{N}}\sum_{n=1}^{N}a_{n} \bigl[R_{n1j}I\{1\leq R_{n1j}\leq c_{n} \}-ER_{n1j}I\{1\leq R_{n1j}\leq c_{n}\}\bigr] \\ &{}+ \frac{1}{b_{N}}\sum_{n=1}^{N}a_{n}R_{n1j}I \{ R_{n1j}> c_{n}\} \\ &{}+ \frac{1}{b_{N}}\sum _{n=1}^{N}a_{n}ER_{n1j}I\{1\leq R_{n1j}\leq c_{n}\} \\ =&I_{1}+I_{2}+I_{3}. \end{aligned}$$

By (2.1) and (2.2), it is easy to show

$$\begin{aligned}& \sum_{n=1}^{\infty}\operatorname{Var}\biggl( \frac{1}{c_{n}}\bigl(R_{n1j}I\{1\leq R_{n1j}\leq c_{n}\} -ER_{n1j}I\{1\leq R_{n1j}\leq c_{n} \}\bigr)\biggr) \\& \quad \leq \sum_{n=1}^{\infty} \frac{1}{c_{n}^{2}}ER_{n1j}^{2}I \{1\leq R_{n1j}\leq c_{n}\} \\& \quad = \sum_{n=1}^{\infty} \frac{m!}{c_{n}^{2}(j-2)!(m-j)!}\sum _{l=0}^{j-2}(-1)^{j-l-2}C_{j-2}^{l} \int_{1}^{c_{n}}\frac {r^{2}}{[l+1+r(m-l-1)]^{2}}\,dr \\& \quad \leq C\sum_{n=1}^{\infty} \frac{1}{c_{n}^{2}} \sum_{l=0}^{j-2} \int_{1}^{c_{n}}1\,dr \leq C\sum _{n=1}^{\infty} \frac{1}{c_{n}}=C\sum _{n=1}^{\infty}\frac {a_{n}}{b_{n}}< \infty, \end{aligned}$$

then by Lemma 2.4, we have

2.7 $$ I_{1}\to0 \quad \mbox{a.s. } n\rightarrow\infty. $$

For any $0<\varepsilon<1$,

$$\begin{aligned}& \sum_{n=1}^{\infty}P\bigl\{ R_{n1j}I \{ R_{n1j}> c_{n}\}>\varepsilon\bigr\} \\& \quad = \sum _{n=1}^{\infty}P\{ R_{n1j}> c_{n}\} = \sum_{n=1}^{\infty} \frac{m!}{(j-2)!(m-j)!}\sum _{l=0}^{j-2}(-1)^{j-l-2}C_{j-2}^{l} \int_{ c_{n}}^{\infty}\frac {1}{[l+1+r(m-l-1)]^{2}}\,dr \\& \quad \leq C \sum_{n=1}^{\infty}\sum _{l=0}^{j-2} \int_{ c_{n}}^{\infty}\frac{1}{r^{2}}\,dr \leq C\sum _{n=1}^{\infty}\frac{1}{c_{n}}=C\sum _{n=1}^{\infty}\frac {a_{n}}{b_{n}}< \infty. \end{aligned}$$

Then by the Borel-Cantelli lemma, we get

2.8 $$ R_{n1j}I\{ R_{n1j}> c_{n}\}\to0 \quad \mbox{a.s. } n\rightarrow\infty. $$

By (2.2) and (2.3), we can obtain

2.9 $$ \limsup_{N\to\infty} \frac{1}{b_{N}}\sum _{n=1}^{N}a_{n}\leq\lambda. $$

Therefore combining (2.8) with (2.9), we can easily conclude

2.10 $$ I_{2}\to0\quad \mbox{a.s. }n\rightarrow\infty. $$

For $I_{3}$, by (2.1) and noting $c_{n}\to\infty$, we get

$$\begin{aligned}& ER_{n1j}I\{1\leq R_{n1j}\leq c_{n}\} \\& \quad = \frac{m!}{(j-2)!(m-j)!}\sum_{l=0}^{j-2}(-1)^{j-l-2}C_{j-2}^{l} \int _{1}^{c_{n}}\frac{r}{[l+1+r(m-l-1)]^{2}}\,dr \\& \quad = \frac{m!}{(j-2)!(m-j)!}\sum_{l=0}^{j-2}(-1)^{j-l-2}C_{j-2}^{l} \frac {1}{(m-l-1)^{2}} \int_{m}^{l+1+c_{n}(m-l-1)}\biggl[\frac{1}{y}- \frac{i+1}{y^{2}}\biggr]\,dy \\& \quad = \frac{m!}{(j-2)!(m-j)!}\sum_{l=0}^{j-2}(-1)^{j-l-2}C_{j-2}^{l} \frac {1}{(m-l-1)^{2}} \\& \qquad {} \cdot\biggl[\log\frac{l+1+c_{n}(m-l-1)}{m}-(i+1) \biggl(\frac{1}{m}- \frac {1}{l+1+c_{n}(m-l-1)}\biggr)\biggr] \\& \quad \sim \frac{m!}{(j-2)!(m-j)!}\sum_{l=0}^{j-2}(-1)^{j-l-2}C_{j-2}^{l} \frac{1}{(m-l-1)^{2}}\log(c_{n}); \end{aligned}$$

then combining with (2.3), we show

2.11 $$ I_{3}\to \frac{\lambda m!}{(j-2)!(m-j)!}\sum _{l=0}^{j-2}C_{j-2}^{l} \frac {(-1)^{j-l-2}}{(m-l-1)^{2}},\quad n\rightarrow\infty. $$

So the proof of (2.4) is completed by combining (2.6), (2.7), (2.10), and (2.11).

By the same argument as in the proof of (2.4), we can get (2.5), so we omit it here. □

#### Remark 2.6

If we take $a_{n}=\frac{(\log n)^{\alpha}}{n}$, $b_{n}=(\log n)^{\alpha +2}$, $\alpha>-2$, it is easy to check that conditions (2.2) and (2.3) hold with $\lambda=\frac{1}{\alpha+2}$, so Theorems 2.1 and 2.2 and 4.1 from Adler [1] are special cases of our Theorem 2.5. There are some other sequences satisfying conditions (2.2) and (2.3), such as (a) $a_{n}=1$, $b_{n}=n^{\beta}$, $\beta>1$, $\lambda=0$; (b) $a_{n}=1$, $b_{n}=n(\log n)^{\gamma}$, $\gamma>1$, $\lambda=0$; (c) $a_{n}=1$, $b_{n}=n(\log n)(\log\log n)^{\delta}$, $\delta>1$, $\lambda =0$; (d) $a_{n}=\frac{(\log\log n)^{\theta}}{n}$, $b_{n}=(\log n)^{2}(\log \log n)^{\theta}$, $\theta\in R$, $\lambda=\frac{1}{2}$, so the conditions (2.2) and (2.3) are mild conditions. At the end of this remark, we point out that only when $a_{n}=L(n)/n$, where $L(n)$ is a slowly varying function, the limit value *λ* will be $\lambda>0$, this is known as an exact strong law, one can refer to Adler [4] for more details. For the weak law, *i.e.*, convergence in probability, one can see Feller [5] for full details.

For $R_{nij}$, $i\geq2$, since the expectation is finite, by the classical strong law of large numbers, we have the following.

#### Theorem 2.7

*For fixed* $m_{n}=m$, *we have for* $2\leq i< j\leq m$,

2.12 $$ \lim_{N\to\infty}\frac{1}{N}\sum_{n=1}^{N} (R_{nij}-ER_{nij})=0\quad \textit{a.s.} $$

### Other limit properties for $R_{nij}$, $2\leq i< j\leq m$

By the above discussion, we know that, for fixed sample size $m_{n}=m$ and $2\leq i< j\leq m$, $\{R_{nij},n\geq1\}$ is a sequence of independent and identically distributed random variables with finite mean, and $L(r)=E(R_{nij}-ER_{nij})^{2}I\{|R_{nij}-ER_{nij}|\leq r\}$ is a slowly varying function at ∞. Therefore the limit properties of $R_{nij}$ for fixed sample size can easily be established by those of the self-normalized sums. We list some of them, such as the central limit theorem (CLT), the law of iterated logarithm (LIL), the moderate deviation principle (MDP), the almost sure central limit theorem (ASCLT). Denote $S_{N}=\sum_{n=1}^{N}(R_{nij}-ER_{nij})$, $V_{N}^{2}=\sum_{n=1}^{N}(R_{nij}-ER_{nij})^{2}$.

#### Theorem 2.8

CLT

*For fixed sample size* $m_{n}=m$, *we know*

2.13 $$ \frac{S_{N}}{V_{N}}\stackrel{\mathrm{{D}}}{\rightarrow}N(0,1). $$

#### Proof

By Theorem 3.3 from Giné *et al.* [6], we can obtain the CLT for $R_{nij}$. □

#### Theorem 2.9

LIL

*For fixed sample size* $m_{n}=m$, *we get*

2.14 $$ \limsup_{N\to\infty}\frac{S_{N}}{V_{N}\sqrt{2\log\log N}}=1\quad \textit{a.s.} $$

#### Proof

By Theorem 1 from Griffin and Kuelbs [7], the LIL for $R_{nij}$ holds. □

#### Theorem 2.10

MDP

*Let* $\{x_{n},n\geq1\}$ *be a sequence of positive numbers with* $x_{n}\to \infty$ *and* $x_{n}=o(\sqrt{n})$, *as* $n\to\infty$, *then*, *for fixed sample size* $m_{n}=m$, *we conclude*

2.15 $$ \lim_{N\to\infty}\frac{1}{x_{N}^{2}}P\biggl\{ \frac{S_{N}}{V_{N}}\geq x_{N}\biggr\} =-\frac{1}{2}. $$

#### Proof

By Theorem 3.1 from Shao [8], we can prove the MDP for $R_{nij}$. □

#### Theorem 2.11

ASCLT

*Suppose that* $0\leq\alpha<1/2$ *and set* $d_{k}=\exp\{(\log k)^{\alpha}\}/k$ *and* $D_{n}=\sum_{k=1}^{n} d_{k}$. *Then*, *for fixed sample size* $m_{n}=m$ *and any* $x\in R$,

2.16 $$ \lim_{k\to\infty}\frac{1}{D_{k}}\sum_{N=1}^{k}d_{N}I \biggl\{ \frac{S_{N}}{V_{N}}\leq x\biggr\} =\Phi(x) \quad \textit{a.s.}, $$

*where* $\Phi(\cdot)$ *is the distribution function of the standard normal random variable*.

#### Proof

By Corollary 1 from Zhang [9], we know ASCLT for $R_{nij}$ holds. □

#### Remark 2.12

It is easy to check that $\eta_{N}/{V_{N}}\stackrel{\mathrm{{p}}}{\rightarrow}1$, then by the Slutsky lemma and Theorem 2.8, we can get Theorem 2.1 from Miao *et al.* [2].

## References

[1] Adler A (2015). Strong laws for ratios of order statistics from exponentials. Bull. Inst. Math. Acad. Sin. (N.S.).

[2] Miao Y, Wang RJ, Adler A (2016). Limit theorems for order statistics from exponentials. Stat. Probab. Lett..

[3] De la Peña VH, Tai TL, Shao QM (2009). Self-Normalized Process: Limit Theory and Statistical Applications.

[4] Adler A (2000). Exact strong laws. Bull. Inst. Math. Acad. Sin..

[5] Feller W (1968). An Introduction to Probability Theory and Its Applications.

[6] Giné E, Götze F, Mason DM (1997). When is the Student *t*-statistic asymptotically standard normal?. Ann. Probab..

[7] Griffin PS, Kuelbs JD (1989). Self-normalized laws of the iterated logarithm. Ann. Probab..

[8] Shao QM (1997). Self-normalized large deviations. Ann. Probab..

[9] Zhang Y (2013). A general result on almost sure central limit theorem for self-normalized sums for mixing sequences. Lith. Math. J..

